# Development and *in vivo* evaluation of intranasal formulations of parathyroid hormone (1-34)

**DOI:** 10.1080/10717544.2021.1889718

**Published:** 2021-03-04

**Authors:** Dan Wang, Yimeng Du, Wenpeng Zhang, Xiaolu Han, Hui Zhang, Zengming Wang, Nan Liu, Meng Li, Xiang Gao, Xiaomei Zhuang, Jing Gao, Aiping Zheng

**Affiliations:** State Key Laboratory of Toxicology and Medical Countermeasures, Beijing Institute of Pharmacology and Toxicology, Beijing, 100850, China

**Keywords:** Parathyroid hormone (1-34), intranasal formulation, permeation enhancer, mucosal toxicity, pharmacokinetics

## Abstract

For efficient intranasal transport of parathyroid hormone (1-34) [PTH(1-34)], there is a great medical need to investigate permeation enhancers for intranasal formulations. In this study, the development of PTH(1-34) intranasal formulations was conducted. Based on conformation and chemical stability studies, the most preferable aqueous environment was determined to be 0.008 M acetate buffer solution (ABS). Subsequently, citric acid and Kolliphor^®^ HS·15 were compared as permeation enhancers. The mechanisms of action of citric acid and Kolliphor^®^ HS·15 were investigated using an *in vitro* model of nasal mucosa, and Kolliphor^®^ HS·15 led to higher permeability of fluorescein isothiocyanate-labeled PTH(1-34) (FITC-PTH) by enhancing both the transcellular and paracellular routes. Moreover, citric acid showed severe mucosal toxicity resulting in cilia shedding, while Kolliphor^®^ HS·15 did not cause obvious mucosa damage. Finally, Kolliphor^®^ HS·15 was studied as a permeation enhancer using a liquid chromatography tandem mass spectrometry (LC-MS/MS) method. The results showed that 5% and 10% Kolliphor^®^ HS·15 increased the bioavailability of PTH(1-34) to 14.76% and 30.87%, respectively. In conclusion, an effective and biosafe PTH(1-34) intranasal formulation was developed by using 10% Kolliphor^®^ HS·15 as a permeation enhancer. Intranasal formulations with higher concentrations of Kolliphor^®^ HS·15 for higher bioavailability of PTH(1-34) could be further researched.

## 1. Introduction

Osteoporosis is a common disease characterized by reduced bone density, leading to an increased risk of fragility fractures of the hip, vertebrae and distal radius. Middle-aged and elderly women are especially susceptible to osteoporosis, and more than 10% of women over 50 have suffered osteoporotic fracture. In China, osteoporosis affects more than 100 million people, with approximately 700,000 osteoporotic hip fractures occurring each year (Dobnig, 2004; Kanis et al., 2012). In the last century, an exciting advance in the treatment of osteoporosis was the introduction of the anabolic osteoporosis drug parathyroid hormone (PTH). PTH can exert osteogenic effects in the human body, resulting in increased bone mass and reversing the deterioration of bone microarchitecture, which accompanies osteoporosis. PTH is an 84-amino acid single-chain polypeptide, and its 1-34 N-terminal fragment, PTH(1-34), has been reported to completely retain its osteogenic activity (Watanabe et al., 2018). In 2002, the first commercial PTH(1-34) product teriparatide (Eli Lilly (United States)) was approved by the Food and Drug Administration (FDA). Subsequently, other PTH(1-34) products were successively developed, such as Forsteo (US PTH 1-34, Eli Lilly), Forteo (EU PTH 1-34, Eli Lilly) and Teribone™ (Japan PTH 1-34, Ashai Kasei).

The therapeutic window of PTH(1-34) for the treatment of osteoporosis is an approximately 24-month period, which requires intermittent administration of PTH(1-34) (Neer et al., 2001). Currently, all commercial PTH(1-34) preparations are injections. Patient compliance with repeated injections is poor, and only 50% of patients continue administration of self-injectable PTH(1-34) for 6 months after commencing treatment. Consequently, it is preferable to develop alternative noninjectable delivery routes for PTH(1-34). Taking advantage of the large surface area and the highly vascularized epithelial layer of the nasal cavity, intranasal administration is considered a promising drug delivery route (Davis, 2001). In comparison with the parenteral route, intranasal administration is noninvasive, amenable to self-medication and virtually painless. Moreover, intranasal administration does not require sterile drug formulations (Dhuria et al., 2010). Compared with oral administration, intranasal administration can avoid the degradation of peptide and protein drugs in the gastrointestinal tract. Moreover, due to the large absorption area, rich blood flow and porous and thin endothelial basement membrane of the nasal cavity, intranasal administration can achieve a much faster onset of action than oral administration. Accordingly, research attention has been focused on evaluating the intranasal delivery of PTH(1-34) for the long-term management of osteoporosis. Several pilot studies provided proof of concept for intranasal PTH(1-34) administration by demonstrating a nasal spray formula of PTH(1-34) to be safe, effective, and well tolerated (Agu et al., 2004; Matsumoto et al., 2006; Sato, 2007).

Drug molecules cross the nasal epithelial cell membrane either by the transcellular route or by the paracellular route, which are both significantly difficult for the delivery of macromolecules. The major hurdle for the development of peptide intranasal administration formulations is their low bioavailability (O’Hagan & Illum, 1990). At present, most intranasal peptide formulations (e.g. desmopressin, salmon calcitonin and nafarelin) are typically devoid of permeation enhancers, resulting in bioavailability lower than 3% (Ozsoy et al., 2009). Consequently, there is a great medical need to investigate permeation enhancers for the efficient intranasal transport of peptide drugs (Pires et al., 2009; Djupesland, 2013; Fortuna et al., 2014; Rohrer et al., 2018). As early as the 1980s, sodium glycocholate and sodium tauro-24,25-dihydrofusidate (STDHF) were reported as permeation enhancers for the intranasal delivery of insulin (Shinichiro et al., 1981; Longenecker et al., 1987). Subsequently, a soybean-derived sterylglucoside (SG) mixture was demonstrated to be a more effective permeation enhancer for nasal absorption of insulin (Maitani et al., 1995). Owing to considerable research efforts on nasal administration of macromolecular drugs, various permeation enhancers, including fusidates, fatty acids, cyclodextrins, ammonium glycyrrhizinate, poly-l-arginine, laureth-9, chitosan, alkylsaccharides, and cyclopenta decalactone (azone), were then reported. However, further clinical application of these permeation enhancers was mainly limited by their mucosal toxicity (Shao & Mitra, 1992; Reardon et al., 1993; Ohtake et al., 2002; Wang et al., 2002; du Plessis et al., 2010).

Despite the potency of intranasally administrated PTH(1-34), the intranasal delivery route is challenging for the 4.1 kDa peptide PTH(1-34), largely because of unfavorable bioavailability. Evidently, it is crucial to adopt a suitable permeation enhancer to effectively and safely promote the absorption of PTH(1-34) across the nasal mucosa. Citric acid has received regulatory approval for pulmonary administration and has been widely studied as a potent absorption enhancer for dry powder inhaler (DPI) formulations of therapeutic macromolecules (Onoue et al., 2009). In recent decades, citric acid has also been reported to be able to accelerate the nasal absorption of therapeutic peptides/proteins (Karasulu et al., 2008). In addition to citric acid, Kolliphor^®^ HS·15, a mixture of mono- and diesters of 12-hydroxystearate (macrogol 15-hydroxystearate) (Brayden et al., 2012), has been investigated as a permeation enhancer for intranasal administration of human growth hormone (hGH) and insulin in animal studies and human trials (Illum, 2012; Lewis et al., 2013). In recent years, Pearson’s group focused on investigating a PTH(1-34) intranasal spray formulation containing Kolliphor^®^ HS·15 as a permeation enhancer (Williams et al., 2018; Pearson et al., 2019). They demonstrated that Kolliphor^®^ HS·15 did not diminish the bioactivity of PTH(1-34), profiled the deposition and clearance of the nasal spray in the nasal cavity, and provided a pharmacokinetic (PK) study. However, a comprehensive study is still needed to investigate the appropriate vehicle, elucidate the action mechanisms of permeation enhancer candidates, and determine the optimal permeation enhancer concentration.

According to the published preclinical data and our preliminary experiments (unpublished), in this manuscript, citric acid and Kolliphor^®^ HS·15 were compared as permeation enhancer candidates for PTH(1-34) intranasal formulations. To probe their permeability-promoting mechanism of action, the effects of Kolliphor^®^ HS·15 and citric acid on the transepithelial electrical resistance (TEER) of the cell monolayer as well as the cellular internalization of PTH(1-34) were investigated. Subsequently, *in vivo* experiments were performed to investigate the nasal mucosal toxicity and PK profiles of the PTH(1-34) intranasal formulations. The ultimate aim of our study was to develop a biosafe PTH(1-34) intranasal formulation with enhanced bioavailability.

## 2. Materials and methods

### 2.1. Materials and instruments

PTH(1-34) was chemically synthesized by Shenzhen Hanyu Pharmaceutical Co., Ltd. (Shenzhen, China). Kolliphor^®^ HS·15 was purchased from BASF (Ludwigshafen, Germany). Sodium deoxycholate was purchased from Dalian Meilun Biotechnology Co., Ltd. (Dalian, China). Milli-Q water was produced using a Milli-Q Plus system (Bedford, MA, USA). Trifluoroacetic acid (TFA) and hyaluronidase were purchased from Sigma-Aldrich. 3-(4,5-Dimethylthiazol-2-yl)-2,5-diphenyltetrazolium bromide (MTT) Kits were purchased from Immutopics Ltd. (San Clemente, CA, USA). Cell culture reagents were from Gibco (Grand Island, NY, USA). All the other chemicals were purchased from the Chinese Medicine Group (Beijing, China).

High-performance liquid chromatography (HPLC) experiments were performed by using a Thermo Ultimate 3000 HPLC system. Microscopic observation experiments were performed using an OLYMPUS BX53 optical microscope. Scanning electron microscopy (SEM) experiments were performed on Hitachi S4800 SEM equipment. Liquid chromatography tandem mass spectrometry (LC-MS/MS) quantification of PTH(1-34) was performed using a Waters Xevo TQ-S triple quadrupole MS system.

### 2.2. Experimental animals

Toads (male) were purchased from the Animal Room of the Academy of Military Medical Sciences (Beijing, China). Healthy male Sprague-Dawley (SD) rats (180 ± 20 g) were purchased from Beijing Weitonglihua Experimental Animal Technology Co., Ltd (Beijing, China). The rats were housed separately in stainless-steel wire cages, maintained in a breeding room at a temperature of 25 ± 1 °C, and given free access to food and water. All of the animal studies were approved by the Animal Ethics Committee at Beijing Institute of Pharmacology and Toxicology (ETHICS CODE Permit Nos. SCXK-(Beijing) 2007-004 and SCXK-(Beijing) 2007-003). Approval was received before the beginning of the animal research. All animal experiments were carried out following the National Institutes of Health Guide for the Care and Use of Laboratory Animals (NIH Publications No. 8023, revised 1978).

### 2.3. HPLC method

HPLC measurements were performed with an HPLC instrument equipped with a photodiode array detector. A C-18 column (Cosmosil 250 × 4.6 mm, 5 µm, Japan) was used, and the sample solutions were applied via an autosampler (Merck-Hitachi, Darmstadt, Germany). Analysis of PTH(1-34) and its degradation products was performed under the following conditions. Mobile phase A was 0.1% (v/v) TFA in acetonitrile, and mobile phase B was 0.1% TFA (v/v) in water. PTH(1-34) was eluted with 26% A/74% B to 46% A/54% B over 10 min and then with 46% A/54% B to 26% A/74% B for 5 min at a flow rate of 1.0 mL/min. A detection wavelength of 214 nm and column temperature of 25 °C were used.

### 2.4. Determination of the aqueous environment

#### 2.4.1. Computational simulations

The starting geometric structure of PTH(1-34) was obtained from 1ET1 in the Protein Data Bank (PDB) database (Illum, 2012). PTH(1-34) was solvated with either 0.063 M phosphate buffer solution (PBS) or 0.032 M acetate buffer solution (ABS) and placed in a 20 × 20 × 20 nm box with periodic boundary conditions. The number of ions in the PBS box or ABS box was determined according to their corresponding concentration and dissociation equilibrium constants. The Gromos 54A7 force field was used (Lewis et al., 2013), and the simulation parameters were references from Automated Force Field Topology Builder (ATB) tools. The dynamics simulations were successively performed as energy minimization, equilibrium under the NPT ensemble and sampling under the canonical (NVT) ensemble. For both of these simulations, the temperature and pressure were kept at 300 K and 1 bar, and the simulation step was 2 fs. The total simulation time for sampling was 300 ns. The molecular dynamics (MD) simulations and analysis of the resulting trajectories were performed by using the GROMACS software package.

#### 2.4.2. Stability study

Stability studies were carried out to clarify the influence of aqueous solvent on the stability of PTH(1-34) during storage and to determine the optimal aqueous environment. First, PBS and ABS were prepared as solvent solutions: 0.063 M PBS was prepared by dissolving 3.73 g sodium dihydrogen phosphate and 36.55 g disodium hydrogen phosphate in 1000 mL Milli-Q water; 0.032 M ABS was prepared as by dissolving 0.664 g glacial acetic acid and 1.64 g sodium acetate trihydrate together in 1000 mL Milli-Q water. Then, 0.016 M ABS, 0.008 M ABS, and 0.004 M ABS were prepared by diluting 0.032 M ABS with Milli-Q water. Following preparation, the pH values of the PBS and ABS solutions were measured using a pH meter (Mettler Toledo FiveEasy Plus).

To examine the stability of peptides in each aqueous medium, 3 mg/mL PTH(1-34) was separately incubated in 0.063 M PBS, 0.032 M ABS, 0.016 M ABS, 0.008 M ABS and 0.004 M ABS. Aliquots of PTH(1-34) were individually incubated at 40 °C, and the PTH(1-34) content was analyzed at 0, 6, 12, 24, 36, 48, 60 and 72 h postincubation. In addition, the impurities of PTH(1-34) in the 0.032 M ABS, 0.016 M ABS, 0.008 M ABS and 0.004 M ABS solutions were quantified by HPLC at the time points of 0, 24, 72 and 120 h postincubation at 40 °C.

### 2.5. Cell study

#### 2.5.1. Cytotoxicity assay

Human nasal epithelial cells (HNEpCs) purchased from the American Type Culture Collection (Rockville, MD, USA) were used as an *in vitro* model of the nasal mucosa. The cells were cultured in culture medium (Eagle’s minimum essential medium (MEM) supplemented with 10% (v/v) fetal bovine serum (FBS) and 1% penicillin/streptomycin solution) and maintained in 5% CO_2_/95% humidified air at 37 °C.

The viability of HNEpCs treated with the permeation enhancer candidates Kolliphor^®^ HS·15 and citric acid was determined by MTT assays. HNEpCs were seeded at a density of 5 × 10^3^ cells/well in 96-well plates (Corning, NY, USA) and cultured for 24 h before the experiment. Kolliphor^®^ HS·15 solutions (in culture median) and citric acid solutions (in culture median) with series concentrations of 0.005%, 0.010%, and 0.015% were applied to the corresponding wells (*n* = 8). The culture medium was used as a negative control, and 20% dimethyl sulfoxide (DMSO) was used as a positive control. Following 6 h of incubation, the test solutions were removed, and the cytotoxicity was determined using a commercial MTT kit. The cells were washed, and then, MTT reagent in culture medium was added for a further 3 h incubation at 37 °C. The remaining media was removed, and 200 μL DMSO was added to each well to dissolve the formazan crystals. The resulting absorbance was read at 490 nm.

#### 2.5.2. Permeability studies

HNEpCs were seeded at a density of 10 × 10^4^ cells/well on Transwell^®^ supports with a pore size of 0.4 μm. TEER (Ω·cm^2^) was determined using a Millicell electrical resistance system (ERS) voltohmmeter (Millipore, Bedford, MA, USA). Cell monolayers with TEER values exceeding 500 Ω·cm^2^ were used for further experiments. Seven test solutions were prepared in culture medium for the permeability study: 10 μg/mL fluorescein isothiocyanate-labeled PTH(1-34) (FITC-PTH) with no permeation enhancer, 10 μg/mL FITC-PTH with 0.005% Kolliphor^®^ HS·15, 10 μg/mL FITC-PTH with 0.010% Kolliphor^®^ HS·15, 10 μg/mL FITC-PTH with 0.015% Kolliphor^®^ HS·15, 10 μg/mL FITC-PTH with 0.005% citric acid, 10 μg/mL FITC-PTH with 0.010% citric acid, and 10 μg/mL FITC-PTH with 0.015% citric acid. Prior to the permeability experiment, the cell monolayers were equilibrated with 1.0 mL Hank’s balanced salt solution (HBSS) in the basolateral compartment and 0.4 mL culture medium in the apical chamber for 30 min. Subsequently, the apical culture medium was withdrawn, and 0.2 mL solution of a test solution was applied. Thereafter, 200 μL receiver samples from the basolateral compartment were taken every 30 min for 4 h, and then, 200 μL fresh HBSS was added to the basolateral compartment. Finally, the FITC-PTH concentration in the collected samples was analyzed using a Varioskan LUX multimode microplate reader with ex/em 485/518 nm.

#### 2.5.3. Effect of permeation enhancers on TEER

HNEpC monolayers were cultured as described above. The baseline TEER was measured before the experiment for 60 min. Kolliphor^®^ HS·15 solutions and citric acid solutions at concentrations of 0.005%, 0.010% and 0.015% were applied to the apical compartment for 2 h, with the TEER recorded at predetermined time points (every 20 min). A chitosan solution at 0.003% w/v in culture medium was used as a positive control. The results for each experiment are presented as percentages relative to their respective baseline TEER.

#### 2.5.4. Cellular internalization of FITC-PTH

The impact of Kolliphor^®^ HS·15 and citric acid on the efficiency of cellular internalization of PTH(1-34) was determined in HNEpCs. HNEpCs were seeded at a density of 1 × 10^5^ cells/well in 24-well plates and cultured for 24 h before the experiment. FITC-PTH solutions (20 μg/mL, in medium) supplemented with Kolliphor^®^ HS·15 or citric acid at concentrations of 0.015%, 0.010% and 0.005% were prepared as test solutions (*N* = 6, *n* = 8). A 20 μg/mL FITC-PTH solution without any permeation enhancer was used as a negative control, and a 20 μg/mL FITC-PTH solution supplemented with 0.1% Triton X-100 was used as a positive control. The test solutions were added to the corresponding wells, and the amount of cellularly internalized PTH was measured after 30 min following sample applications. At the end of the incubation, the test solutions were removed, and the cells were washed with PBS 7 times. Finally, the cells were lysed with cell lysis buffer. The resulting lysis products were collected for fluorescence measurement and protein content measurement. The fluorescence measurement was carried out using a Varioskan LUX multimode microplate reader with ex/em 485/518 nm, and the protein content was measured by using a commercial BCA protein assay kit (Solarbio Life Sciences). The amount of internalized PTH(1-34) in each well was normalized to the protein content.

### 2.6. Nasal mucosal toxicity study of permeation enhancers

#### 2.6.1. Study of mucosa morphology

Mucosa morphology was considered a direct indicator of nasal mucosal toxicity. The permeation enhancer solutions, including 0.2% citric acid solution, 0.4% citric acid solution, 0.6% citric acid solution, 5% Kolliphor^®^ HS·15 solution, 10% Kolliphor^®^ HS·15 solution and 15% Kolliphor^®^ HS·15 solution, were prepared by dissolving citric acid or Kolliphor^®^ HS·15 into 0.008 M ABS. In addition, saline was studied as a negative control, and a 1% sodium deoxycholate water solution was studied as a positive control. This mucosa morphology study was performed *in vivo* using healthy male SD rats. The rats were lightly anesthetized with diethyl ether and intranasally administered test solutions. Generally, 0.1 mL of the corresponding solution was equally administered to the two nostrils of one rat for 15 consecutive days. Finally, 2 days after stopping intranasal administration, the rats were sacrificed by an intraperitoneal injection of urethane and their hearts were perfused with saline. The nasal septum mucosae were then carefully peeled off, fixed with 3% w/v glutaraldehyde (diluted by PBS), and dehydrated with a gradient of ethanol. Finally, the fixed nasal mucosae were dried at the CO_2_ critical point, sprayed with ion sputtering, and observed under SEM.

#### 2.6.2. Study of cilia movement

The effect of 10% Kolliphor^®^ HS·15 on nasal mucosa cilia movement was further studied by using toad palate mucosa. Saline and 1% w/v sodium deoxycholate solution were used as negative and positive controls, respectively. Briefly, 3 × 5 mm of mucosae was removed from the upper jaw of sacrificed toads (30–40 g, male and female). The mucosae were washed with saline and treated by dropwise addition of 0.5 mL of 10% Kolliphor^®^ HS·15 solution, saline or 1% w/v sodium deoxycholate solution (*n* = 6). After dripping, the mucosae were observed under a microscope, and the time from the application of the test solution to the cessation of ciliary movement was recorded as the lasting time of cilia movement (LTCM). Then, the mucosae were washed with saline 5 times to rinse off the applied test solutions. After washing, the mucosae were observed under a microscope, and the time from washing finished until cilia movement restarted was recorded as restoring LTCM.

### 2.7. *In vivo* pharmacokinetic study

#### 2.7.1. *In vivo* pharmacokinetic study

The intranasal PTH(1-34) formulations were prepared according to Table 1. A PTH(1-34) subcutaneous injection was also prepared since the PTH(1-34) bioavailability of intranasal formulations needs to be calculated relative to the subcutaneously administered PTH(1-34). The subcutaneous injection F_S_ comprising 0.03 mg/mL PTH(1-34) was prepared by dissolving 15 mg PTH(1-34) powder into 500 mL 0.008 M ABS. For the *in vivo* PK study, freshly prepared PTH(1-34) formulations were used.

**Table 1.** Intranasal formulations and subcutaneous injection of PTH(1-34).

Formulation group	Formulation name	Administration route	PTH(1-34) (mg/mL)	Kolliphor^®^ HS·15 (w/v)	Solvent
Group-Subcutaneous	F_S_	Subcutaneous	0.03 mg/mL	/	0.008 M ABS
Group-Intranasal	F_N1_	Intranasal	3 mg/mL	0%	0.008 M ABS
	F_N2_	Intranasal	3 mg/mL	5%	0.008 M ABS
	F_N3_	Intranasal	3 mg/mL	10%	0.008 M ABS

Healthy male SD rats were randomly divided into four groups to be administered PTH(1-34) formulations (Table 1). For subcutaneous administration, the rats received a subcutaneous dose of 178.6 μg/kg PTH(1-34) in formulation F_S_. For intranasal administration, the rats were sedated with isoflurane vapor, and then, the liquid formulation (F_N1_, F_N2_ or F_N1_) was equally administered to the two nostrils at a dose of 5.358 μg/kg. After administration of PTH(1-34), 0.4 mL blood samples were taken from each rat via a jugular vein cannula at 0 (immediately after administration), 5, 10, 15, 30, 60, 90, 120 and 180 min postadministration. Generally, each blood sample was collected into a heparinized tube and then immediately centrifuged at 5,000 rpm for 20 min (4 °C). The resultant supernatant plasma was stored at −20 °C until it was analyzed by LC-MS/MS.

#### 2.7.2. LC-MS/MS method

A stock solution of 10 ng/mL PTH(1-34) was prepared in blank rat plasma. The stock solution was serially diluted with blank rat plasma to generate standard solutions with PTH(1-34) concentrations of 8, 4, 2, 1, 0.5, 0.25, 0.18 and 0 ng/mL. All standard solutions were freshly prepared before the LC-MS/MS measurement experiment.

The plasma samples were frozen at −20 °C prior to LC-MS/MS measurement. Before the experiment, the frozen plasma samples were thawed at room temperature. For each sample, 150 μL plasma was transferred into a 1.5 mL centrifuge tube, and 1 mL crash solvent A (75:25:0.1 acetonitrile:H_2_O:formic acid v/v) was added. The centrifuge tube was vortexed and then centrifuged at 4000 rpm for 15 min at 4 °C. After centrifugation, 900 μL supernatant was transferred to a new 1.5 mL centrifuge tube, and 50 μL crash solvent B (60:34:5:1 acetonitrile:H_2_O:TFE/TFA v/v) plus 50 μL Milli-Q water was added to this tube. The tube was vortexed and then centrifuged at 4000 rpm for 15 min at 4 °C. Finally, the supernatant was transferred into a sample bottle for LC-MS/MS analysis. LC-MS/MS measurements were performed according to a previously published method (Kay et al., 2015). The standard solutions for the LC-MS/MS analysis were prepared by the same procedure as described above. The PTH(1-34) concentration of the plasma samples was defined according to the standard curve obtained from the PTH(1-34) standard solutions.

### 2.8. Statistical analysis

In this manuscript, the statistical analysis was performed with the software package SPSS (v.16.0.2, SPSS, Chicago, IL, USA), and the data are presented as the mean ± standard deviation.

## 3. Results

### 3.1. Determination of the aqueous environment for the PTH(1-34) liquid formulation

**Figure 1.**
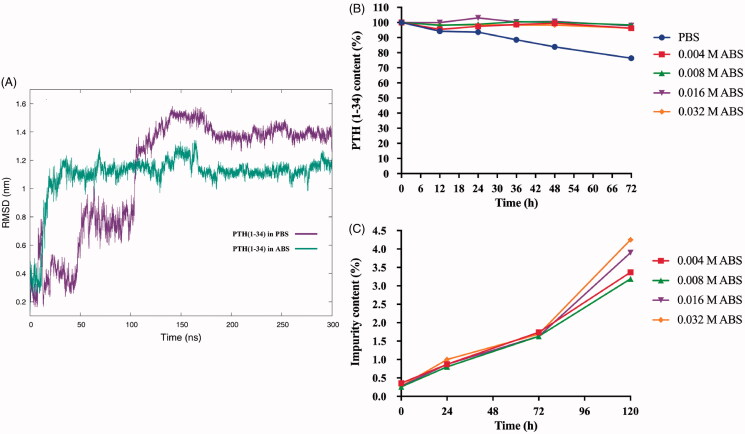
(A) The evolution of the root mean square deviation (RMSD) of PTH(1-34) with respect to the minimized initial structure of PTH(1-34) simulations. HPLC measurement results of (B) the PTH(1-34) content and (C) the impurities in the stability study of PTH(1-34) PBS and ABS-based solutions.

When developing peptides for medical use, considerations of their conformational and chemical stability under formulation environments are essential. First, MD simulations were used to understand the conformational equilibria of polypeptides in solution. The root mean square deviation (RMSD) with respect to the initial structure for PTH(1-34) in 0.063 M PBS and 0.032 M ABS as a function of time is reported in Figure 1(A). Generally, in ABS, the RMSD value remained steady at approximately 1.2 nm after an increase at the beginning. This result revealed that the PTH(1-34) molecule tended to be stable at a constant conformation. In contrast to the ABS environment, in PBS, large deviations of RMSD occurred during the whole 300 ns of simulation, indicating that the PTH(1-34) molecule tended to populate a wide range of conformational states. Furthermore, the chemical stability of PTH(1-34) in PBS or ABS was studied based on HPLC analysis. Generally, all PTH(1-34) solutions were stored at 40 °C, resulting in time-dependent degradation of PTH(1-34). The HPLC measurement results in Figure 1(B) show that the content of PTH(1-34) in the PBS-based solution significantly decreased to 76.34% after 72 h of incubation. On the other hand, there was no detectable decrease in the PTH(1-34) content in any of the ABS-based solutions. Furthermore, the impurities in the ABS-based PTH(1-34) solutions were measured. Figure 1(C) clearly shows that differences among the four ABS solutions became evident at 120 h, and 0.008 M ABS exhibited the lowest amount of impurities. This result demonstrated that PTH(1-34) had optimal stability in 0.008 M ABS solution. The PTH(1-34) formulations in the subsequent studies were all prepared based on 0.008 M ABS.

### 3.2. Cytotoxicity study

**Figure 2.**
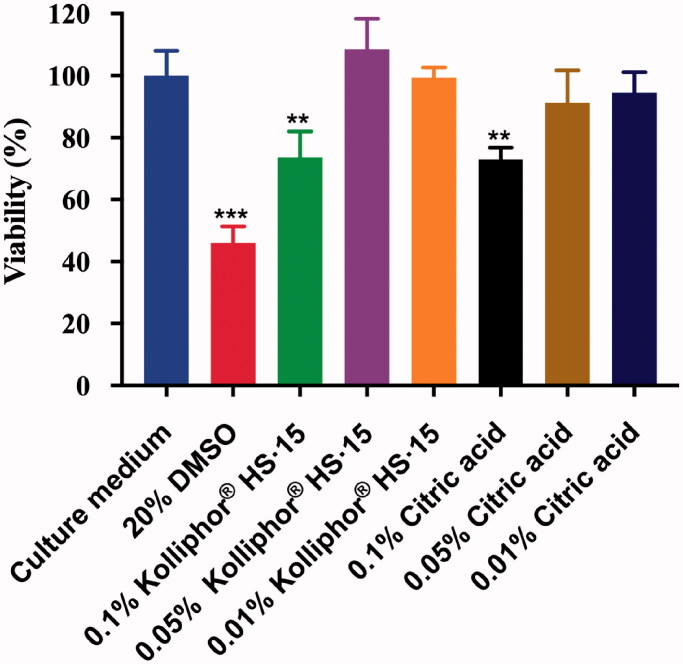
Effect of Kolliphor^®^ HS·15 and citric acid on HNEpC viability (6 h). (*p < .05; **p < .01; ***p < .001, compared culture medium, *n* = 8).

The cytotoxic effects of citric acid and Kolliphor^®^ HS·15 were assessed on HNEpCs, and the results are summarized in Figure 2. With 20% DMSO (positive control), the viability was statistically lower than that of the culture medium (negative control), indicating significant cytotoxicity. In the MTT assay, the viability following 6 h of exposure to Kolliphor^®^ HS·15 or citric acid at concentrations of 0.01% and 0.05% was comparable with that of the negative control (culture medium). However, at the higher concentration of 0.1%, both Kolliphor^®^ HS·15 and citric acid exhibited obvious cytotoxicity, with 73.67% and 73.03% viability, respectively. The treatment of HNEpCs with citric acid and Kolliphor^®^ HS·15 induced similar concentration-dependent cytotoxicity.

### 3.3. Effect of Kolliphor^®^ HS·15 and citric acid on the permeability of FITC-PTH across HNEpC layers

**Figure 3.**
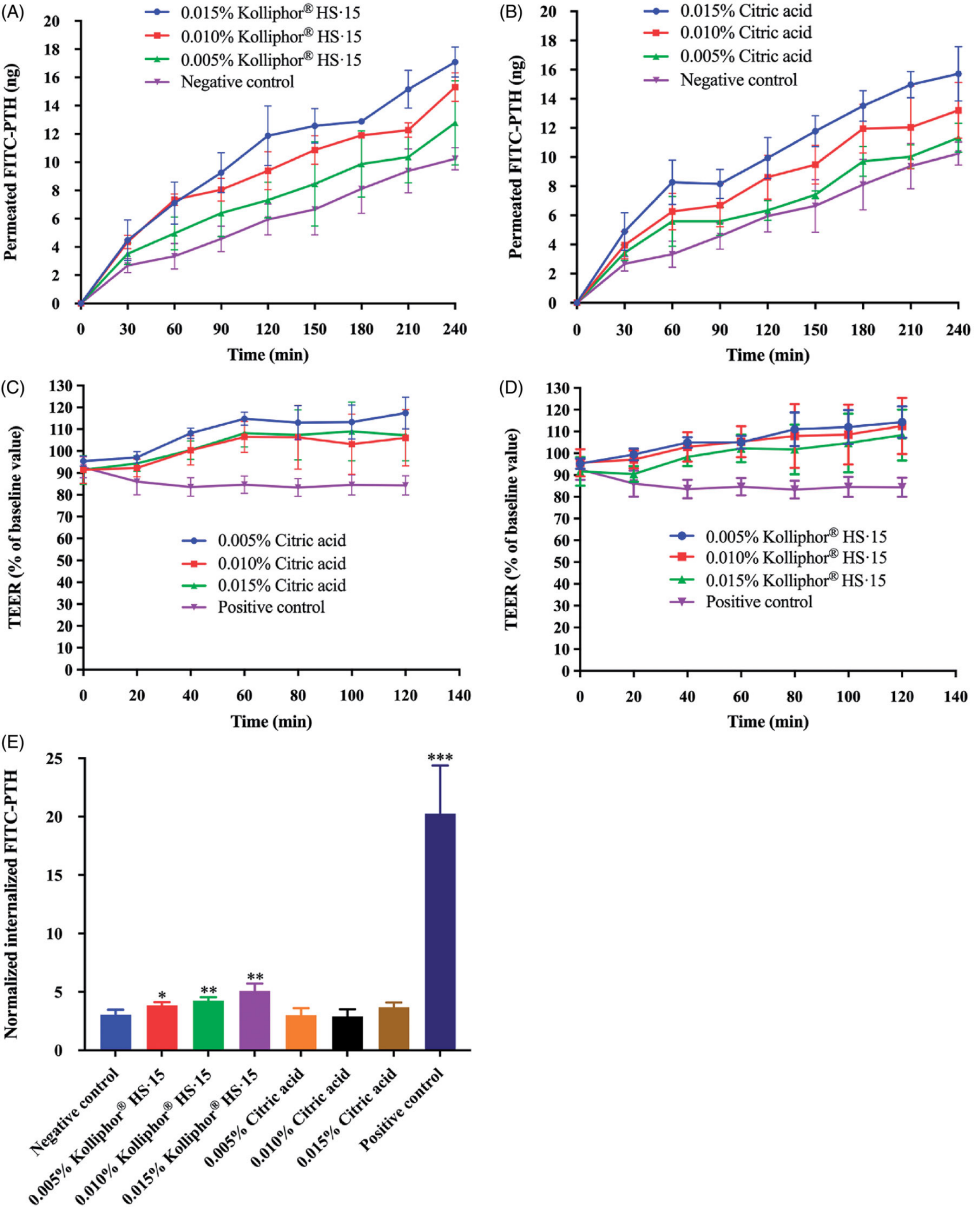
Profile of permeated FITC-PTH vs time for formulations with (A) Kolliphor^®^ HS·15 and (B) citric acid. Reduction in TEER after apical-side application of (C) Kolliphor^®^ HS·15 and (D) citric acid (*n* = 8). (E) Normalized cellular internalized FITC-PTH for 60 min incubation. (*p < .05; **p < .01; ***p < .001, compared culture medium, *n* = 8).

The impact of Kolliphor^®^ HS·15 and citric acid on the permeability of FITC-PTH across the HNEpC layers is illustrated in Figure 3(A,B) as permeated FITC-PTH vs time profiles. For the Kolliphor^®^ HS·15 group (Figure 3(A)), the permeation profile displayed a clear correlation between the duration of the permeation-enhancing effect and the concentration of Kolliphor^®^ HS·15. Specifically, 0.015% and 0.010% Kolliphor^®^ HS·15 caused a significantly increased permeation rate for 120 min and 60 min, respectively, while 0.005% Kolliphor^®^ HS·15 caused a slightly increased permeation rate for only 30 min. By contrast, the profiles for the citric acid group (Figure 3(B)) showed an enhanced permeation rate at all tested concentrations (0.015%, 0.010% and 0.005%) over 60 min relative to the control group, whereby the degree of enhancement (slope of the curve) was generally dependent on the concentration of citric acid.

To investigate the opening of the tight junction of HNEpC monolayers subjected to permeation enhancers, TEER was recorded following the application of Kolliphor^®^ HS·15 and citric acid solutions. The results are presented as percentages relative to the respective baseline TEER values (Figure 3(C,D)). Following apical application of the Kolliphor^®^ HS·15 and citric acid solutions, a reduction in the TEER was observed immediately. Then, TEER reversal to the baseline level was detected at the 40 min time point for all citric acid solutions. For the Kolliphor^®^ HS·15 solutions, the reversal time points were 40 min for the 0.015% solution and 20 min for the 0.010% and 0.005% solutions. On the other hand, with chitosan solution (positive control) treatment, the TEER values were lower relative to the baseline value at 0 min over 120 min. These results suggested that both Kolliphor^®^ HS·15 and citric acid can transiently open intercellular tight junctions, thus increasing the permeability of PTH(1-34) through the paracellular route. Subsequently, the effect of Kolliphor^®^ HS·15 and citric acid on the internalization of FITC-PTH by cells was investigated. Clearly, 0.1% Triton X-100, which increased the permeability of the cell membrane, significantly promoted the cellular internalization of FITC-PTH. Similarly, the presence of Kolliphor^®^ HS·15 led to the promotion of cellular internalization in a concentration-dependent manner. In contrast, citric acid did not exhibit an obvious promotion effect. These findings suggested that Kolliphor^®^ HS·15 is able to enhance the transcellular permeability of PTH(1-34), while citric acid is not.

### 3.4. Nasal mucosal toxicity study

**Figure 4.**
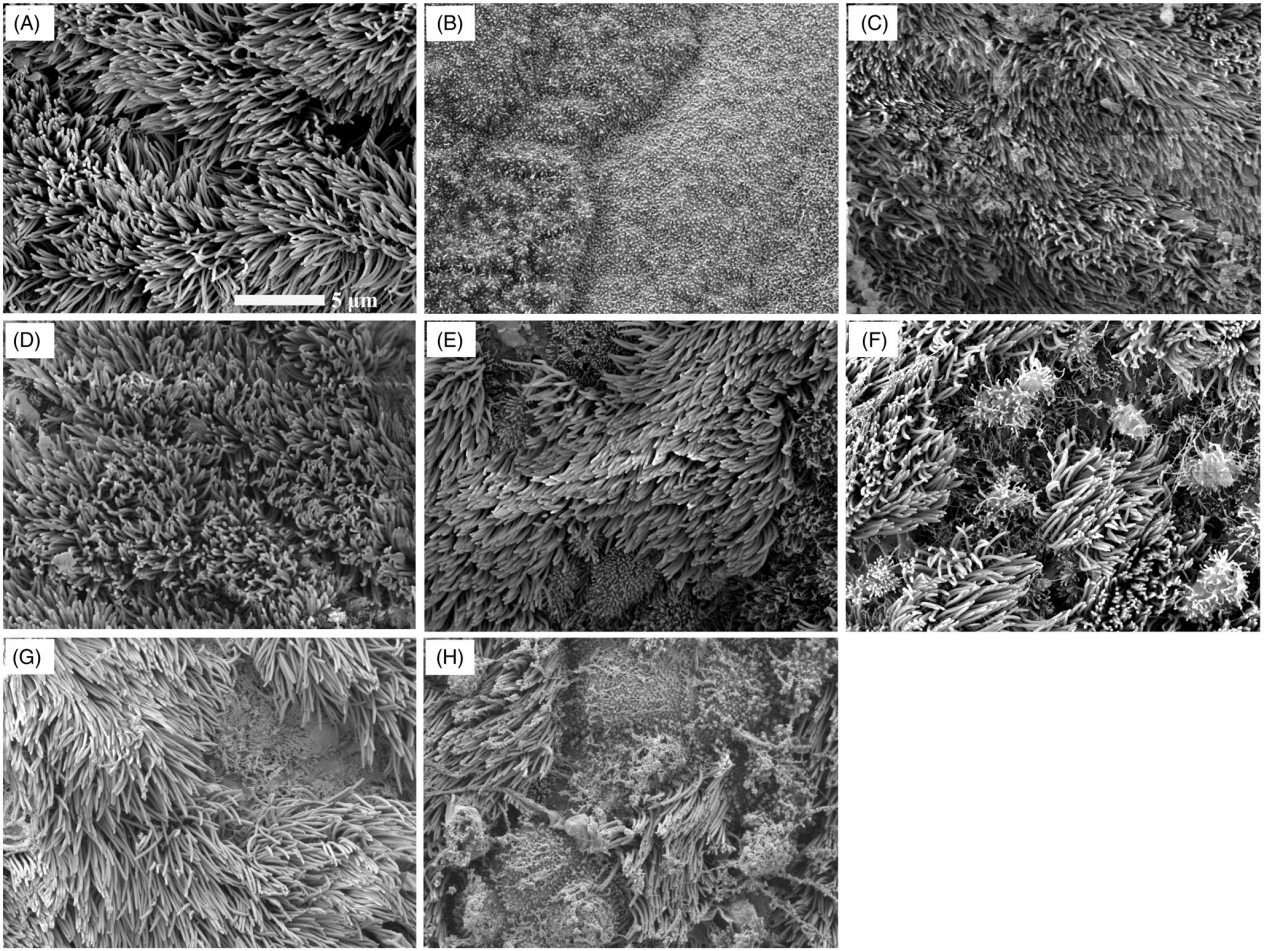
SEM images of rat mucosae treated with (A) saline, (B) 1% sodium deoxycholate, (C) 5% Kolliphor^®^ HS·15, (D) 10% Kolliphor^®^ HS·15, (E) 15% Kolliphor^®^ HS·15, (F) 0.2% citric acid, (G) 0.4% citric acid and (H) 0.6% citric acid.

The safety of permeation enhancers is particularly important for clinical applications. Therefore, the nasal mucosal toxicity of the permeation enhancer candidates Kolliphor^®^ HS·15 and citric acid was studied. First, the most direct evaluation method of nasal mucosal toxicity is to observe the mucosal morphology after administration of permeation enhancer solutions. As shown in Figure 4(A), the cilia of the nasal mucosa treated with saline were dense and neatly oriented. However, the nasal mucosa treated with 1% sodium deoxycholate (Figure 4(B)) was seriously damaged, with all of the cilia shedding and leaving only the exposed base of cilia. The 5% and 10% Kolliphor^®^ HS·15 solutions (Figure 4(C,D)) did not cause any obvious damage, with neatly arranged cilia and no cilia shedding observed on the treated mucosa. This result demonstrated the biocompatibility of partial shedding. However, the 15% Kolliphor^®^ HS·15 solution (Figure 4(E)) resulted in partial cilia shedding. On the other hand, all three citric acid solutions (Figure 4(F–H)) caused mucosal damage, and the severity of the damage appeared increase with increasing concentration.

The influence of Kolliphor^®^ HS·15 on the movement of cilia was further investigated by using saline as a negative control and 1% sodium deoxycholate as a positive control. The continuous swing time of cilia in normal saline was 725.50 ± 114.32 min (Table 2). In contrast, the cilia of the nasal mucosa treated with 1% sodium deoxycholate all fell off, resulting in the LTCM being 0 min. The LTCM for the nasal mucosa treated with 10% Kolliphor^®^ HS·15 was 74.25 ± 10.69 min, and after washing away the Kolliphor^®^ HS·15 solution, the ciliary swing recovered after 85.5 ± 71.93 min. Clearly, Kolliphor^®^ HS·15 can inhibit ciliary swing, but with a possibility of recovery.

**Table 2.** Effects of different excipients on ciliary movement (*n* = 4).

Sample	LTCM (min)	Restoring LTCM (min)
Physiological saline	725.50 ± 114.32	/
10% Kolliphor^®^ HS·15	74.25 ± 10.69	85.5 ± 71.93
1% Sodium deoxycholate	0	0

### 3.5. *In vivo* study of permeation enhancer candidates

**Figure 5.**
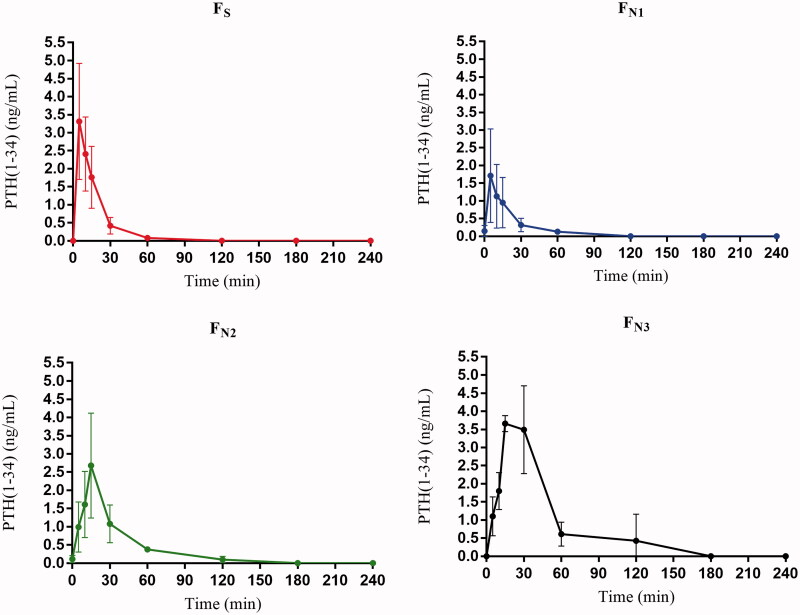
*In vivo* PK profiles after administration of the subcutaneous injection F_S_ and intranasal formulations F_N1_, F_N2_ and F_N3_ (*n* = 6).

The *in vivo* PK profile of the PTH(1-34) formulations is shown in Figure 5, and the corresponding PK parameters are presented in Table 3. For the subcutaneous injection of PTH(1-34), the profile showed rapid absorption (mean T_max_ of 5 min) and elimination (mean T_1/2_ of 10.15 min), and the PTH(1-34) level nearly returned to zero at 60 min postinjection. As a reference, a permeation enhancer-free intranasal formulation F_N1_ was investigated as a control sample. The profile for F_N1_ exhibited rapid absorption with T_max_ of 5 min but slower elimination with T_1/2_ of 15.93 min. The bioavailability of PTH(1-34) for F_N1_ calculated relative to F_S_ was 6.27%. This low bioavailability clearly demonstrated that, as a macromolecule, PTH(1-34) was poorly absorbed through the intranasal route. The performance of the intranasal formulations F_N2_ and F_N3_ containing 5% and 10% Kolliphor^®^ HS·15 as permeation enhancers was then studied. Both formulations had a T_max_ of 15 min, indicating slower absorption. However, formulation F_N3_ exhibited an obviously different profile, with a wider peak and significantly slower elimination (T_1/2_ of 28.25 min). The bioavailability of F_N2_ and F_N3_ was calculated to be 14.76% and 30.87%, respectively. Evidently, Kolliphor^®^ HS·15 significantly enhanced the intranasal permeation of PTH(1-34).

**Table 3.** PK parameters of the subcutaneous injection F_S_ and intranasal formulations F_N1_, F_N2_ and F_N3_ (*n* = 6).

Formulation	T_1/2_ (min)	T_max_ (min)	C_max_ (ng·mL^-1^)	AUC_0-240_ (ng·min·mL^-1^)	F (%)
F_S_	10.15 ± 3.86	5	3.31 ± 1.61	56.86 ± 26.15	/
F_N1_	15.93 ± 8.57	5	1.72 ± 1.32	32.19 ± 23.66	6.27
F_N2_	16.66 ± 2.64	15	2.68 ± 1.44	81.67 ± 13.44	14.76
F_N3_	28.25 ± 14.37	15	3.66 ± 0.22	170.96 ± 53.21	30.87

## 4. Discussion

The essential point for the development of a liquid PTH(1-34) formulation was ensuring the stability of PTH(1-34). In this context, the aqueous environment was screened by utilizing MD simulations and HPLC analysis. In recent decades, MD simulations have been increasingly reported to investigate the conformational equilibria of polypeptides in solution (Colombo et al., 2002). In this article, we present MD simulations of PTH(1-34) in PBS and ABS, indicating that the conformation of PTH(1-34) is more stable in ABS than in PBS. The HPLC analysis results further confirmed that PTH(1-34) had the best chemical stability in 0.008 M ABS. According to our previous capillary electrophoresis (CE) analysis, the isoelectric point (pI) of PTH(1-34) is approximately pH 7.77, indicating easy solubility and stability under acidic condition. The pH values of PBS and ABS were 7.4 and 4.0, respectively. Consequently, 0.008 M ABS had the best stability performance, most likely due to its acidic pH value and low ionic strength. Similarly, the nine-amino acid peptide desmopressin has been reported to be more stable at pH values between 4.5 and 5.5 (Law et al., 2003). In conclusion, 0.008 M ABS was determined to be the most preferable aqueous environment for the development of PTH(1-34) liquid formulations.

It is essential to develop an intranasal permeation enhancer with a low toxic effect on the nasal mucosa, especially for chronic therapy. In our study, the toxicity profiles of Kolliphor^®^ HS·15 and citric acid were studied both in cell cultures and in preclinical animal models. Cytotoxicity experiments showed that at the same concentration, Kolliphor^®^ HS·15 and citric acid exhibited similar cytotoxicity; however, they had apparently different mucosal toxicity in the preclinical animal model (SD rat). In a pilot study, the biocompatibility of intranasally administered Kolliphor^®^ HS·15 was demonstrated over a 5-day period in a rat model (Illum, 2012). In our study, biocompatibility was investigated by dosing rat nasal mucosa with Kolliphor^®^ HS·15 and citric acid solutions over 15 consecutive days. During intranasal administration of citric acid solutions, bleeding of the nose was observed in the tested rats. In contrast, the application of Kolliphor^®^ HS·15 at much higher concentrations (5% and 10%) did not cause any morphological damage to the nasal mucosa and did not induce any observed discomfort in the rats. The difference in toxicity in cell cultures and in animal models has been reported in previous permeation enhancer studies (Shubber et al., 2015). Likewise, in our study, Kolliphor^®^ HS·15 and citric acid exhibited similar cytotoxicity, but Kolliphor^®^ HS·15 showed significantly lower local toxicity in the *in vivo* nasal mucosa. In summary, Kolliphor^®^ HS·15 demonstrated a promising toxicity profile for subsequent development of intranasal formulations.

The absorption of macromolecule drugs through nasal administration is mainly limited by poor permeation across the nasal membrane (O’Hagan & Illum, 1990). In previous studies, the permeation behavior of macromolecule drugs across nasal mucosa and the efficiency of permeation enhancers have been widely investigated *in vitro* by utilizing an artificial cellulose membrane, Nephrophan, or excised animal nasal mucosa. However, the inherent defects of the *in vitro* membranes made them unsuitable for mimicking the *in vivo* nasal membrane permeation behavior. First, the cellulose membrane limits the permeation of drug molecules only by their pore size without any complex biological structures. In comparison with that through the cellulose membrane, the penetration of drug molecules through the nasal mucosa was much lower due to the complex composition of the mucosa. Moreover, the lipophilic nasal mucosa is more permeable to unionized drugs, while the hydrophilic cellulose membrane is more permeable to ionic drugs (Illum, 2002). All these differences make cellulose membranes unsuitable for mimicking nasal mucosa. To utilize excised animal nasal mucosa, the freshness of mucosa greatly influenced the drug permeation behavior. It has been reported that the drug permeation efficiency was significantly lower in refrigerated mucosa than in freshly excised mucosa (Fabrizio et al., 2009). In addition, *in vitro* experiments caused nasal mucosa to suffer prolonged hypoxia and consequential activity alterations. In summary, *in vitro* membrane permeation experiments cannot represent the real *in vivo* situation, and thus, an *in vitro* and *in vivo* correlation cannot be accomplished. In our study, instead of an *in vitro* membrane permeation study, the permeation-enhancing effect of Kolliphor^®^ HS·15 and citric acid was investigated in *in vitro* cell models. Furthermore, the PTH(1-34) intranasal formulations were evaluated through *in vivo* experiments using a preclinical model of SD rats.

Kolliphor^®^ HS·15 and citric acid at concentrations of 0.005%, 0.010% and 0.015% were compared to enhance the permeation of FITC-PTH across HNEpC monolayers. These studied concentrations were lower than the maximum concentration (0.05%) in the cytotoxicity experiment. Figure 3(A,B) clearly show that Kolliphor^®^ HS·15 exhibited a prolonged permeation-enhancing effect at higher concentrations, while citric acid did not present such a concentration-dependent action time. This difference may be attributed to their different action mechanisms. The permeation-enhancing effect on the transcellular transport and paracellular transport of PTH was investigated. First, the TEER was recorded under treatment with Kolliphor^®^ HS·15 and citric acid solutions to indicate the opening of cell tight junctions. Chitosan is a classical permeability enhancer that opens epithelial tight junctions. Comparison of TEER vs time profiles for the test solutions and chitosan illustrates significant differences in their behaviors. Chitosan maintained the TEER at a lower level during a 2-h exposure. Regarding Kolliphor^®^ HS·15 and citric acid, the TEER decreased immediately after application of the test solutions and then gradually recovered to baseline over approximately 40 min. This recovery profile illustrated that the epithelial cells can ‘fix’ the opened tight junctions without removal of the permeation enhancers, resulting in a transient permeation-enhancing effect through the paracellular route. Subsequently, the transcellular route was evaluated by measuring the amount of cellular internalized FITC-PTH to reflect the permeability of the cell membrane to PTH(1-34). As a nonionic surfactant, Kolliphor^®^ HS·15 monomers can be incorporated into the cell membrane to enhance membrane permeability by perturbing the packing order of phospholipids within the cell membrane bilayer (Suwalsky et al., 2006; Busch & Unruh, 2011). Our experiment demonstrated that Kolliphor^®^ HS·15 clearly promoted the intracellular internalization of FITC-PTH, implying that Kolliphor^®^ HS·15 has the ability to enhance the transcellular transport of PTH(1-34) across nasal epithelial cells. On the other hand, citric acid did not exhibit any enhancement of the transcellular transport of PTH(1-34). In summary, in addition to the transient enhancement of the paracellular route, Kolliphor^®^ HS·15 can enhance the transcellular route by increasing cell membrane permeability. It is reasonable to speculate that the transcellular route is the main reason for the concentration-dependent action time.

Currently, immunoassays are considered the gold-standard approach for bioanalytical assays. However, the immunoassay-based measurement of PTH(1-34) suffered cross-reactivity to similar compounds of the target peptide (Satterwhite et al., 2010). For example, in previous clinical studies, a radioimmunoassay (RIA) aimed at detecting PTH(1-34) was also capable of detecting other PTH species, including PTH(1-84), PTH(1-31), PTH(2-34) and PTH(3-34) (Satterwhite et al., 2010). In recent years, the LC-MS/MS method has been reported to be more applicable to peptide quantitation without being subject to the abovementioned cross-reactivity issues (Neubert et al., 2013; Chappell et al., 2014). In our preliminary studies, the matrix effects introduced by rat plasma were compared by ELISA and LC-MS/MS. Unfortunately, measurable absorbance signals were occasionally detected in ELISA measurements of blank plasma due to hemolysis, residence of blood cells and hyperlipidemia of the tested rat. On the other hand, the blank plasma tested by LC-MS/MS always presented a signal much lower than the lower limit of quantitation (LLOQ). Apparently, LC-MS/MS is a more robust quantification method, and our following *in vivo* PK study was carried out using LC-MS/MS.

The formulation F_N3_ containing 10% Kolliphor^®^ HS·15 had higher PTH(1-34) bioavailability than the formulation F_N2_ containing 5% Solutol^®^ HS·15. This finding is consistent with the concentration-dependent manner of the permeation enhancement profile in the cellular study (Figure 3(A)). The T_max_ of the formulations containing Kolliphor^®^ HS·15 (F_N2_ and F_N3_) was clearly delayed compared with the formulation without Kolliphor^®^ HS·15 (F_N1_). This outcome is most likely due to the time required for Kolliphor^®^ HS·15 to insert into the cell membrane of the *in vivo* nasal mucosa. In addition, ciliary movement observations demonstrated that Kolliphor^®^ HS·15 inhibited ciliary movement in a reversible way. The inhibition of ciliary movement can inhibit cilia clearance in the PTH(1-34) formulations and thus increase the residence time of liquid formulations in the nasal cavity. This effect can explain the wide peak in the PK profile of formulation F_N3_. The F_N3_ solution remaining on the nasal mucosa served as a reservoir, and thus, the PTH(1-34) molecules could continue permeating across the nasal mucosa at a high level from the ‘reservoir’ for a several-minute period. It is reasonable to deduce that by taking advantage of the synergistic action of the inhibition of ciliary movement, opening of the tight junctions and enhancement of the cellular membrane permeability, Kolliphor^®^ HS·15 achieved its significant permeation-enhancing effect. In summary, our study demonstrated an optimal PTH(1-34) intranasal formulation (F_N3_) in 0.008 M ABS supplemented with 10% Kolliphor^®^ HS·15 as a permeation enhancer. This formulation provided 30.87% bioavailability of PTH(1-34) in a safe manner.

Kolliphor^®^ HS·15 has a promising future and has been previously reported as a permeation enhancer to promote macromolecular drugs crossing the nasal and intestinal mucosa in phase I clinical trials (Illum et al., 2012). Based on the *in vivo* PK data in our study, the intranasal formulation supplemented with Kolliphor^®^ HS·15 represents an effective alternative formulation for PTH(1-34) injection. As a typical model, rats have been frequently used for drug delivery via nasal administration of peptides (Illum et al., 2012). With the aim of developing a liquid PTH(1-34) nasal spray, our continued research will evaluate the formulation in a large animal model.

## 5. Conclusion

Overall, this study aimed to develop an intranasal formulation of PTH(1-34). The research work first determined the optimal aqueous environment to be 0.008 M ABS, exhibiting preferable conformation and chemical PTH(1-34) stability. Citric acid and Kolliphor^®^ HS·15 were investigated as permeation enhancers. Due to severe mucosal toxicity, formulations containing citric acid were excluded from promising intranasal formulations. On the other hand, Kolliphor^®^ HS·15 exhibited good biosafety and significant permeation enhancement in *in vivo* studies. An intranasal formulation of PTH(1-34) with 10% Kolliphor^®^ HS·15 produced high bioavailability (30.87%) in a preclinical animal model. In conclusion, this intranasal formulation is an attractive alternative to the injection formulation of PTH(1-34). It is worthwhile to promote future research on Kolliphor^®^ HS·15-based intranasal PTH(1-34) formulations, including a comprehensive evaluation of the formulation, investigation of slightly higher Kolliphor^®^ HS·15 concentrations and even advancement to early clinical trails.
